# Modifiable lifestyle factors and severe COVID-19 risk: a Mendelian randomisation study

**DOI:** 10.1186/s12920-021-00887-1

**Published:** 2021-02-03

**Authors:** Shuai Li, Xinyang Hua

**Affiliations:** 1 Centre for Epidemiology and Biostatistics, Melbourne School of Population and Global Health, The University of Melbourne, 207 Bouverie Street, Parkville, VIC 3010 Australia; 2 Centre for Cancer Genetic Epidemiology, Department of Public Health and Primary Care, University of Cambridge, Cambridge, UK; 3 Precision Medicine, School of Clinical Sciences at Monash Health, Monash University, Clayton, VIC Australia; 4 Centre for Health Policy, Melbourne School of Population and Global Health, The University of Melbourne, Parkville, VIC Australia; 5 The National Perinatal Epidemiology Unit, Nuffield Department of Population Health, University of Oxford, Oxford, UK

**Keywords:** COVID-19, Lifestyle factors, Mendelian randomisation, Causation assessment, Obesity, Smoking, Alcohol consumption, Physical activity

## Abstract

**Background:**

Lifestyle factors including obesity and smoking are suggested to be correlated with increased risk of COVID-19 severe illness or related death. However, whether these relationships are causal is not well known; neither for the relationships between COVID-19 severe illness and other common lifestyle factors, such as physical activity and alcohol consumption.

**Methods:**

Genome-wide significant genetic variants associated with body mass index (BMI), lifetime smoking, physical activity and alcohol consumption identified by large-scale genome-wide association studies (GWAS) of up to 941,280 individuals were selected as instrumental variables. Summary statistics of the genetic variants on severe illness of COVID-19 were obtained from GWAS analyses of up to 6492 cases and 1,012,809 controls. Two-sample Mendelian randomisation analyses were conducted.

**Results:**

Both per-standard deviation (SD) increase in genetically predicted BMI and lifetime smoking were associated with about two-fold increased risks of severe respiratory COVID-19 and COVID-19 hospitalization (all P < 0.05). Per-SD increase in genetically predicted physical activity was associated with decreased risks of severe respiratory COVID-19 (odds ratio [OR] = 0.19; 95% confidence interval [CI], 0.05, 0.74; P = 0.02), but not with COVID-19 hospitalization (OR = 0.44; 95% CI 0.18, 1.07; P = 0.07). No evidence of association was found for genetically predicted alcohol consumption. Similar results were found across robust Mendelian randomisation methods.

**Conclusions:**

Evidence is found that BMI and smoking causally increase and physical activity might causally decrease the risk of COVID-19 severe illness. This study highlights the importance of maintaining a healthy lifestyle in protecting from COVID-19 severe illness and its public health value in fighting against COVID-19 pandemic.

## Background

Obesity and smoking are well-known health-related lifestyle factors. Studies have reported the correlations between obesity and severe illness or related death of COVID-19 [1–3]. For smoking, its relationship with the risk of severe COVID-19 is controversial: some studies suggest that current smokers might have a lower risk [2, 4–6]. However, the Centre for Disease Control and Prevention suggests that both people with obesity and smoking are at increased risk of COVID-19 severe illness [7]. The relationships mentioned above are mainly suggested by observational studies, which of themselves are subject to bias, provide low level of evidence and have limited ability in supporting causality. For other lifestyle factors, such as physical activity and alcohol consumption, little is known about if they are associated with severe illness of COVID-19. A study has found that accelerometer-measured physical activity was associated with decreased likelihood of being a COVID-19 patient in the UK Biobank; no association was found for accelerometer-measured physical activity with the likelihood of death related to COVID-19, or for self-reported physical activity with the likelihoods of being a COVID-19 patient or of death related to COVID-19 [8].

Mendelian randomisation (MR) uses exposure-associated genetic variants as instrumental variables to assess the causality between exposures and outcomes [9]. As genetic variants are randomly allocated at conception, MR resembles a randomised controlled trial and is less subject to confounding than observational studies. The publicly available genome-wide association studies (GWAS) summary statistics provide valuable resources for assessing the causality between lifestyle factors and the risk of COVID-19 severe illness.

MR studies have been conducted to investigate the causality of body mass index (BMI), smoking and physical activity with COVID-19 risk. Inconsistent results have been found for BMI [8, 10, 11], a causal effect with limited precision has been found for smoking [10], and no evidence of causality was found for physical activity [8]. However, the sample sizes of these studies could have limited the abilities of these studies in detecting causal evidence and obtaining precise causal effect estimates.

This study aimed to investigate the causality between four lifestyle factors, namely BMI, smoking, physical activity and alcohol consumption, and severe illness of COVID-19 using a large sample size and the two-sample MR approach [12].

## Methods

### COVID-19 data source

Summary-level data were obtained from two GWAS analyses conducted by the COVID-19 Host Genetic Initiative [13] (Release 4 in September 2020): (1) 2972 very severe respiratory confirmed COVID-19 cases, which were defined as hospitalized laboratory confirmed SARS-CoV-2 infection (RNA and/or serology based) with death or respiratory support, and hospitalization with COVID-19 as primary reason for admission, compared with 284,472 population controls; and (2) 6492 hospitalized confirmed COVID-19 cases, which were defined as hospitalized laboratory confirmed SARS-CoV-2 infection (RNA and/or serology based) and hospitalization due to corona-related symptoms, compared with 1,012,809 controls. The majority (≥ 90%) of the participants included in the GWAS analyses were of European ancestry. Details of the GWAS analyses can be found at https://www.covid19hg.org/.

### Genetic instrumental variables to lifestyle factors

Genome-wide significant genetic variants identified from GWAS were selected as instrumental variables for the investigated lifestyle factors. This study only used independent sentinel genetic variants found from primary analysis of relevant GWAS; genetic variants identified from conditional analysis, i.e., secondary signals, were not used.

- BMI: 656 variants with P < 10^–8^ (Additional file 1: Supplementary Table 1) identified from the meta-analysis of up to 681,275 individuals of European ancestry from the Genetic Investigation of ANthropometric Traits consortium and UK Biobank, explaining ~ 7.0% variation in BMI [14].
- Smoking: 126 variants with P < 5 × 10^–8^ (Additional file 1: Supplementary Table 2) identified from the GWAS of up to 462,690 individuals from the UK Biobank for a lifetime smoking measure capturing smoking initiation, heaviness and duration, explaining 1.3% variation in the lifetime smoking measure [15].
- Physical activity: five variants with P < 5 × 10^–8^ (Additional file 1: Supplementary Table 3) identified to be associated with accelerometer-measured overall physical activity (measured as average vector magnitude) in a sample of up to 91,105 UK Biobank individuals, explaining ~ 0.2% variation in overall physical activity [16].
- Alcohol consumption: 81 variants with P < 5 × 10^–8^ (Additional file 1: Supplementary Table 4) identified to be associated with standard drinks per week in a sample of up to 941,280 individuals of European ancestry from the GWAS and Sequencing Consortium of Alcohol and Nicotine use, explaining ~ 0.6% variation in alcohol consumption measured as drinks per week [17].

Proxies with a minimum linkage disequilibrium r^2^ = 0.8 were used for two (Additional file 1: Supplementary Table 1), two (Additional file 1: Supplementary Table 3) and one (Additional file 1: Supplementary Table 4) variants of BMI, physical activity and alcohol consumption, respectively, that were unavailable in the COVID-19 data sources. One alcohol consumption variant had no proxy available, so it was not included in analysis.

**Table 1:** Odds ratios (OR) and 95% confidence intervals (CI) of the genetically predicted lifestyle factors with COVID-19 severe illness

Lifestyle factor	Number of variants	Severe respiratory COVID-19		COVID-19 hospitalization	
		OR (95% CI)	P	OR (95% CI)	P
Body mass index	656	1.91 (1.55, 2.35)	7.4 × 10^–10^	1.75 (1.52, 2.01)	9.0 × 10^–15^
Lifetime smoking	126	1.84 (1.08, 3.13)	0.02	2.15 (1.52, 3.03)	1.5 × 10^–05^
Physical activity	5	0.19 (0.05, 0.74)	0.02	0.44 (0.18, 1.07)	0.07
Alcohol consumption: all variants	80	1.64 (0.70, 3.81)	0.25	1.57 (0.90, 2.73)	0.11
Alcohol consumption: rs1229984, rs2532276 removed	78	0.64 (0.25, 1.64)	0.35	1.11 (0.57, 2.17)	0.76

OR and 95% CI were expressed as per standard deviation increase in genetically predicted levels in body mass index, lifetime smoking, accelerometer-measured physical activity and alcohol consumption (log-transformed standard drinks per week)

### Statistical analyses

The statistical power was calculated using the proportion of variation in the lifestyle factor explained by the genetic instrumental variables, the sample sizes of the COVID-19 GWAS, and the method proposed by Burgess [18]. From the formula (12) of Burgess, the causal effect size *β*, log-OR per-standard deviation (SD) increase in the genetically predicted lifestyle factor, can be detected with 80% statistical power at the significance level of 0.05 is

$$\beta =\frac{{\Phi }^{-1}\left(0.8\right)+1.96}{{\rho }_{GX}\sqrt{N{\mathbb{P}}\left(Y=1\right){\mathbb{P}}\left(Y=0\right)}}$$

where Φ^−1^ is the inverse of the cumulative distribution function of the standard normal distribution, *ρ*_*GX*_^*2*^ is the proportion of variation in the lifestyle risk factor explained by the genetic instrumental variables, *N* is the sample size of the COVID-19 GWAS, and *ℙ*(*Y* = 1) and *ℙ*(*Y* = 0) are the proportions of cases and controls, respectively, in the COVID-19 GWAS. The values of the parameters used in the calculation for each lifestyle factor can be found in Additional file 2: Supplementary Table 5.

The main analyses were performed using inverse-variance weighted (IVW) method under a random-effects model [19], which assumes that all genetic variants are valid instrumental variables, or any horizontal pleiotropy, i.e., genetic variants were associated with COVID-19 risk through pathways other than the investigated lifestyle factor, must be balanced. The reported odds ratios (ORs) on COVID-19 risk were for per-SD increase in the genetically predicted value in BMI, lifetime smoking measure, accelerometer-measured physical activity and alcohol consumption (log-transformed standard drinks per week).

Leave-one-out analyses, i.e., applying IVW after removing each genetic variant in turn, were performed to assess if the results were driven by any single variant. If there were any, relevant genetic variants were removed and ORs were estimated again.

Sensitivity analyses were performed using MR-Egger regression [20], weighted median method [21] and weighted mode method [22], which relax MR assumptions and allow some genetic instrumental variables to be invalid, but are less powerful than IVW method. MR-Egger regression can provide consistent causal effect estimates even all genetic instrumental variables are invalid, while weighted median and weighted mode methods can provide consistent causal effect estimates when up to 50% genetic instrumental variables are invalid. The more consistency across the point estimates of the methods, the greater the evidence supporting the causal effect of the investigated lifestyle factor on COVID-19 severe illness. The overall directional pleiotropy of the genetic instrumental variables, i.e., unbalanced horizontal pleiotropy, can be detected by the intercept of the MR-Egger regression, which is expected to be different from zero if directional pleiotropy exists.

The analyses were conducted using the TwoSampleMR R package [23]. All statistical tests were two-sided. Results with a nominal P value < 0.05 were considered statistically significant.

## Results

For BMI, lifetime smoking, physical activity and alcohol consumption, respectively, this study has 80% statistical power at the significance level of 0.05 to detect a per-SD OR of 1.22, 1.57, 3.23 and 1.96 or greater on severe respiratory COVID-19, and an per-SD OR of 1.14, 1.36, 2.21 and 1.58 or greater on COVID-19 hospitalization.

Table 1 shows the causal effect estimates using IVW method. Both genetically predicted BMI and lifetime smoking were found to be associated with increased risk of COVID-19 severe illness: the per-SD OR of genetically predicted BMI was 1.91 (95% confidence interval [CI], 1.55, 2.35, P = 7.4 × 10^–10^) for severe respiratory COVID-19 and 1.75 (95% CI 1.52, 2.01; P = 9.0 × 10^–10^) for COVID-19 hospitalization; the per-SD OR of genetically predicted lifetime smoking was 1.84 (95% CI, 1.08 to 3.13, P = 0.02) for severe respiratory COVID-19 and 2.15 (95% CI 1.52, 3.03; P = 1.5 × 10^–5^) for COVID-19 hospitalization. Genetically predicted physical activity was found to be associated with decreased risk of severe respiratory COVID-19 (per-SD OR = 0.19; 95% CI 0.05, 0.74; P = 0.02), but not with COVID-19 hospitalization (per-SD OR = 0.44; 95% CI 0.18, 1.07; P = 0.07), though most of the 95% CI did not include one. No evidence of association was found for genetically predicted alcohol consumption with severe respiratory COVID-19 or COVID-19 hospitalization (both P > 0.1). There was evidence of heterogeneity between the genetic variants of BMI and alcohol consumption, respectively, but not between those of lifetime smoking or physical activity (Table 2).

**Table 2:** Heterogeneity test results for the genetic variants

Lifestyle factor	Method	Q	df	P
*Severe respiratory COVID-19*				
Body mass index	Inverse variance weighted	776.073	655	7.4 × 10^–04^
Body mass index	MR-Egger	770.065	654	1.1 × 10^–03^
Lifetime smoking	Inverse variance weighted	137.387	125	0.21
Lifetime smoking	MR-Egger	137.335	124	0.19
Physical activity	Inverse variance weighted	3.273	4	0.51
Physical activity	MR-Egger	3.005	3	0.39
Alcohol consumption: all variants	Inverse variance weighted	118.041	79	2.9 × 10^–03^
Alcohol consumption: all variants	MR-Egger	110.051	78	0.01
Alcohol consumption: rs1229984, rs2532276 removed	Inverse variance weighted	99.353	77	0.04
Alcohol consumption: rs1229984, rs2532276 removed	MR-Egger	99.280	76	0.04
*COVID-19 hospitalization*				
Body mass index	Inverse variance weighted	772.906	655	9.7 × 10^–04^
Body mass index	MR-Egger	771.083	654	1.0 × 10^–03^
Lifetime smoking	Inverse variance weighted	131.212	125	0.33
Lifetime smoking	MR-Egger	131.192	124	0.31
Physical activity	Inverse variance weighted	3.629	4	0.46
Physical activity	MR-Egger	1.852	3	0.60
Alcohol consumption: all variants	Inverse variance weighted	117.782	79	3.1 × 10^–03^
Alcohol consumption: all variants	MR-Egger	115.557	78	3.7 × 10^–03^
Alcohol consumption: rs1229984, rs2532276 removed	Inverse variance weighted	108.280	77	0.01
Alcohol consumption: rs1229984, rs2532276 removed	MR-Egger	108.188	76	0.01

From the leave-one-out analyses, similar OR estimates were found for BMI (Additional files 4, 5: Supplementary Figs. 1 and 2), lifetime smoking (Additional files 6, 7: Supplementary Figs. 3 and 4) and physical activity (Additional files 8, 9: Supplementary Figs. 5 and 6), suggesting that the observed associations for these three genetically predicted lifestyle factors were unlikely to be driven by any single genetic variant outlier. Genetic variants rs1229984 and rs2532276 appeared to be substantially influence the OR estimates in the analyses for alcohol consumption with both severe respiratory COVID-19 (Additional file 10: Supplementary Fig. 7) and COVID-19 hospitalization (Additional file 11: Supplementary Fig. 8). After removing the two variants, genetically predicted alcohol consumption had a per-SD OR = 0.64 (95% CI 0.25, 1.64; P = 0.35) with severe respiratory COVID-19, and a per-SD OR = 1.11 (95% CI 0.57, 2.17; P = 0.76) with COVID-19 hospitalization (Table 1). Although weak evidence of heterogeneity between the remaining genetic variants were still found (Table 2), the leave-one-out analyses did not suggest there were significant outliers left (Additional files 12, 13: Supplementary Figs. 9 and 10). The two variants were removed from subsequent analyses.

**Fig. 1:**
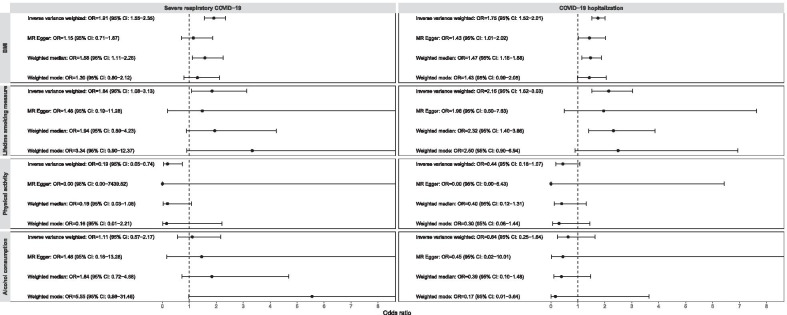
Odds ratios (OR) and 95% confidence intervals (CI) of the genetically predicted lifestyle factors with COVID-19 severe illness across Mendelian randomisation methods. OR and 95% CI were expressed as per standard deviation increase in genetically predicted levels in body mass index (BMI), lifetime smoking measure, accelerometer-measured physical activity and alcohol consumption (log-transformed standard drinks per week). The plots were right-truncated to better present the confidence intervals

Although there was evidence of heterogeneity between the genetic variants of BMI (Table 2), the tests of MR-Egger regression intercepts suggested there was no evidence of overall directional pleiotropy of the genetic variants on COVID-19 hospitalization (P = 0.21), and a weak evidence in the analysis for severe respiratory COVID-19 (Intercept = 0.009; 95% CI 0.001, 0.018; P = 0.02; Table 3). These were also supported by the MR funnel plots, which was generally symmetrical for COVID-19 hospitalization and little asymmetrical for severe respiratory COVID-19 (Additional files 14, 15: Supplementary Figs. 11 and 12). No evidence of overall directional pleiotropy was found for the genetic variants of the other three lifestyle factors (all P > 0.18). The MR funnel plots for lifetime smoking (Additionals file 16, 17: Supplementary Figs. 13 and 14) and alcohol consumption (Additional files 18, 19: Supplementary Figs. 15 and 16) were generally symmetrical, while the plots for physical activity could not properly be assessed as there were five genetic variants only (Additional files 20, 21: Supplementary Figs. 17 and 18). From the sensitivity analyses, causal effect estimates with consistent directions were found across the MR-Egger regression, weighted median method and weighted mode method for all the investigated lifestyle factors, though some estimates were with wider 95% CIs (Fig. 1).

**Table 3:** Test results for the MR-Egger regression intercepts

Lifestyle factor	Intercept estimate	Intercept standard error	P
*Severe respiratory COVID-19*			
Body mass index	0.010	0.004	0.02
Lifetime smoking	0.003	0.016	0.83
Physical activity	0.108	0.210	0.64
Alcohol consumption: rs1229984, rs2532276 removed	0.004	0.018	0.81
*COVID-19 hospitalization*			
Body mass index	0.004	0.003	0.21
Lifetime smoking	0.001	0.010	0.89
Physical activity	0.162	0.096	0.19
Alcohol consumption: rs1229984, rs2532276 removed	-0.003	0.013	0.80

## Discussion

Using a two-sample MR approach, we found evidence that BMI has a causal effect on increased risk of COVID-19 severe illness, same as the findings by Ponsford et al. [10] and Leong et al. [11], but different from those by Zhang et al. [8]. Our causal effect estimates were of greater precision, in terms of the width of the confidence interval (log-OR scale), than those found by Ponsford et al., e.g., the per-SD OR on COVID-19 hospitalization estimated by Ponsford et al. was 1.47 (95% CI 1.18, 1.83) with a confidence interval width of 0.44, while we estimated it to be 1.75 (95% CI 1.52, 2.01) with a confidence interval width of 0.28. The difference is likely to be due to that we used the summary statistics from COVID-19 GWAS analyses of larger sample sizes; Ponsford et al. used the summary statistics from a GWAS of severe COVID-19 with respiratory failure including 1610 cases and 2205 controls [24], and the Release 3 data from the COVID-19 Host Genetic Initiative including 3199 cases and 897,488 controls in the GWAS of COVID-19 hospitalization. Leong et al. [11] also used the Release 3 data from the COVID-19 Host Genetic Initiative but reported the OR as per unit increase in BMI, e.g., 1.12 (95% CI 1.03, 1.13) for COVID-19 hospitalization; therefore, their results cannot be directly compared with ours. The MR study by Zhang et al. [8] reported null associations between genetically predicted BMI and COVID-19 outcomes including being a COVID-19 patient and death related to COVID-19, different from the outcomes investigated in our study. Zhang et al. used the UK Biobank data, the sample size of which might not provide sufficient statistical power to detect an association.

The finding for BMI is consistent with the correlation between obesity and severe illness observed in COVID-19 patients [1–3]. Obesity is plausible to contribute to COVID-19 severe illness. Featured with increased macrophage infiltration that associated with abnormal production of pro-inflammatory cytokines and insulin secretory, obesity contributes to systemic immune dysregulation [25–27], which could contribute to abnormal immune response to the coronavirus SARS-CoV-2 that results severe illness [28].

We found evidence that lifetime smoking has a causal effect on increased risk of COVID-19 severe illness, same as the findings by Ponsford et al. investigating the same lifetime smoking measure [10]. Similar to the results for BMI, our causal effect estimates for lifetime smoking were of greater precision than those found by Ponsford et al. For comparison, the per-SD OR on COVID-19 hospitalization estimated by Ponsford et al. was 4.27 (95% CI 2.10, 8.65) with a confidence interval width of 1.42, while we estimated it to be 2.15 (95%: 1.52, 3.03) with a confidence interval width of 0.69. In COVID-19 patients, smoking has not been found to be associated with the risk of death [2], and lower than expected prevalence of smoking has even been observed [4–6]. However, these studies considered smoking as a binary (current, non-current smokers) or categorical (current, former, never smokers) variable only without any consideration on smoking heaviness or duration. In addition, there might be misclassification in the collected smoking behaviours for COVID-19 patients, especially in emergency contexts. The lifetime smoking measure used by our study is more accurate to reflect the cumulative exposure to smoking, and it has been validated to be associated with lung cancer and coronary heart disease through MR analyses [15].

For the first time, we provided evidence that physical activity causally decreases the risk of severe respiratory COVID-19. Physical activity studied in this study is measured by accelerometer [16]; therefore, it is more accurate than self-reported one. The MR study by Zhang et al. [8] using UK Biobank data reported a null association for either accelerometer-measured or self-reported physical activity; however, similar to their analyses for BMI, they studied different COVID-19 outcomes and the sample size might not be sufficient. Nevertheless, only five genetic instrumental variables were used by our study, and they explained ~ 0.2% variation in accelerometer-measured physical activity only; the estimates for the causal effects were not of great precision. Furthermore, although the MR-Egger regression intercept was not different from zero, it had a considerable magnitude with a suggestion that there might be horizontal pleiotropy of the physical activity genetic instrumental variables. The association could be driven by BMI, as there was a negative genetic correlation between physical activity and BMI [16].

For alcohol consumption, although we found evidence that there were two outlier genetic variants influencing the association estimates using all genetic variants and removed the two variants, null associations were found. The null associations suggest that alcohol consumption might not change the risk of COVID-19 severe illness. However, the null associations might also be due to statistical power; our study did not have sufficient power to detect small effect sizes as the observed ORs of 1.64 and 1.11 with severe respiratory COVID-19 and COVID-19 hospitalization, respectively.

BMI, smoking and physical activity are modifiable, so they could be targeted to reduce severe illness of COVID-19. This study highlights the importance of maintaining a healthy lifestyle in protecting from COVID-19 severe illness. The findings also have a profound public health value—a healthy lifestyle could be helpful for fighting against the COVID-19 pandemic.

Limitations of this study included that there might be bias in the causal effect estimates, as there was some sample overlapping between the lifestyle factors GWAS and COVID-19 GWAS, e.g., UK biobank participants were included in the GWAS of COVID-19 hospitalization. However, the bias might not be substantial, because UK Biobank contributed 413 cases (6.4% of the total cases) only to and 99.9% UK Biobank participants were controls in the GWAS of COVID-19 hospitalization (no UK Biobank participants were included in the GWAS of severe respiratory COVID-19; see Additional file 3: Supplementary Table 6), with an implication that the vast majority of the overlapping samples were likely to be controls in the GWAS of COVID-19; an unbiased causal effect estimate is expected if the associations between risk factor and genetic instrumental variables are obtained from controls [29]. Another limitation is that the findings might not be applicable to populations of non-European ancestry, as the exposure and outcome summary statistics for the genetic instrumental variables were from GWAS of participants almost entirely of European ancestry. Our main analysis included eight statistical tests, but we did not perform multiple testing adjustment; however, the five associations for BMI, lifetime smoking and physical activity with nominal P < 0.05 were still significant even assessed using the false discovery rate (FDR; all FDR < 0.04).

## Conclusions

This two-sample MR study finds evidence that BMI and smoking causally increase and physical activity might causally decrease the risk of COVID-19 severe illness, with an implication that maintaining a healthy lifestyle could protect from severe illness of COVID-19.

## Supplementary Information

**Additional file 1:** Supplementary Tables 1–4. Summary statistics for genetic instrumental variables of the four lifestyle factors.

**Additional file 2:** Supplementary Table 5. Parameter values used in statistical power analysis.

**Additional file 3:** Supplementary Table 6. Sample sizes of participating studies in the COVID-19 Host Genetic Initiative GWAS Release 4 in September 2020.

**Additional file 4:** Supplementary Fig. 1. Leave-one-out analysis results for body mass index and severe respiratory COVID-19.

**Additional file 5:** Supplementary Fig. 2. Leave-one-out analysis results for body mass index and COVID-19 hospitalization.

**Additional file 6:** Supplementary Fig. 3. Leave-one-out analysis results for lifetime smoking and severe respiratory COVID-19.

**Additional file 7:** Supplementary Fig. 4. Leave-one-out analysis results for lifetime smoking and COVID-19 hospitalization.

**Additional file 8:** Supplementary Fig. 5. Leave-one-out analysis results for physical activity and severe respiratory COVID-19.

**Additional file 9:** Supplementary Fig. 6. Leave-one-out analysis results for physical activity and COVID-19 hospitalization.

**Additional file 10:** Supplementary Fig. 7. Leave-one-out analysis results for alcohol consumption and severe respiratory COVID-19 using all genetic variants.

**Additional file 11:** Supplementary Fig. 8. Leave-one-out analysis results for alcohol consumption and COVID-19 hospitalization using all genetic variants.

**Additional file 12:** Supplementary Fig. 9. Leave-one-out analysis results for alcohol consumption and severe respiratory COVID-19 after removing rs1229984 and rs2532276.

**Additional file 13:** Supplementary Fig. 10. Leave-one-out analysis results for alcohol consumption and COVID-19 hospitalization after removing rs1229984 and rs2532276.

**Additional file 14:** Supplementary Fig. 11. Funnel plot for body mass index and severe respiratory COVID-19.

**Additional file 15:** Supplementary Fig. 12. Funnel plot for body mass index and COVID-19 hospitalization.

**Additional file 16:** Supplementary Fig. 13. Funnel plot for lifetime smoking and severe respiratory COVID-19.

**Additional file 17:** Supplementary Fig. 14. Funnel plot for lifetime smoking and COVID-19 hospitalization.

**Additional file 18:** Supplementary Fig. 15. Funnel plot for alcohol consumption and severe respiratory COVID-19 after removing rs1229984 and rs2532276.

**Additional file 19:** Supplementary Fig. 16. Funnel plot for alcohol consumption and COVID-19 hospitalization after removing rs1229984 and rs2532276.

**Additional file 20:** Supplementary Fig. 17. Funnel plot for physical activity and severe respiratory COVID-19.

**Additional file 21:** Supplementary Fig. 18. Funnel plot for physical activity and COVID-19 hospitalization.

## Data Availability

This study used publicly available genome-wide association study summary statistics. The summary statistics for lifestyle factors are reported by Refs. 14–17. The summary statistics for COVID-19 risk are the Release 4 in September 2020 from the COVID-19 Host Genetic Initiative, which can be accessed at https://www.covid19hg.org/results/. No permissions are required to access these data.

## Abbreviations

BMI: Body mass index
CI: Confidence interval
FDR: False discovery rate
GWAS: Genome-wide association study
IVW: Inverse-weighted variance
MR: Mendelian randomisation
OR: Odds ratio
SD: Standard deviation
