# Supplementary material for: Modifiable lifestyle factors and severe COVID-19 risk: a Mendelian randomisation study
Source: BMC Med Genomics. 2021 Feb 3;14:38. doi: 10.1186/s12920-021-00887-1 (PMC7856619; doi:10.1186/s12920-021-00887-1)
Supplement: Supplementary file 2 — Additional file 2: Supplementary Table 5. Parameter values used in statistical power analysis. [file 12920_2021_887_MOESM2_ESM.docx]

**Supplementary Table 5. Parameter values used in statistical power analysis**

| Lifestyle factor | ρ_GX_^2^ | N | ℙ(Y = 1) | ℙ(Y = 0) |
| --- | --- | --- | --- | --- |
| Severe respiratory COVID-19 | | | | |
| Body mass index | 0.070 | 287444 | 0.01 | 0.99 |
| Lifetime smoking | 0.013 | 287444 | 0.01 | 0.99 |
| Physical activity | 0.002 | 287444 | 0.01 | 0.99 |
| Alcohol consumption | 0.006 | 287444 | 0.01 | 0.99 |
| COVID-19 hospitalization | | | | |
| Body mass index | 0.070 | 1019301 | 0.006 | 0.994 |
| Lifetime smoking | 0.013 | 1019301 | 0.006 | 0.994 |
| Physical activity | 0.002 | 1019301 | 0.006 | 0.994 |
| Alcohol consumption | 0.006 | 1019301 | 0.006 | 0.994 |
