# Supplementary material for: Modifiable lifestyle factors and severe COVID-19 risk: a Mendelian randomisation study
Source: BMC Med Genomics. 2021 Feb 3;14:38. doi: 10.1186/s12920-021-00887-1 (PMC7856619; doi:10.1186/s12920-021-00887-1)
Supplement: Supplementary file 3 — Additional file 3: Supplementary Table 6. Sample sizes of participating studies in the COVID-19 Host Genetic Initiative GWAS Release 4 in September 2020. [file 12920_2021_887_MOESM3_ESM.docx]

**Supplementary Table 6. Sample sizes of participating studies in the COVID-19 Host Genetic Initiative GWAS Release 4 in September 2020**

| Study | Number of cases | Number of controls |
| --- | --- | --- |
| Severe respiratory COVID-19 GWAS | | |
| Amsterdam UMC COVID study group | 66 | 1413 |
| Biobanque Quebec COVID19 | 55 | 480 |
| Determining the Molecular Pathways and Genetic Predisposition of the Acute Inflammatory Process Caused by SARS-CoV-2 | 101 | 302 |
| FinnGen | 54 | 238657 |
| GEN-COVID | 468 | 2472 |
| Genes & Health | 24 | 27353 |
| Genetic determinants of COVID-19 complications in the Brazilian population | 450 | 1637 |
| genomiCC | 1676 | 8380 |
| The genetic predisposition to severe COVID-19 | 78 | 3778 |
| COVID-19 hospitalization GWAS | | |
| Amsterdam UMC COVID study group | 108 | 1413 |
| Ancestry | 250 | 1967 |
| Biobanque Quebec COVID19 | 181 | 354 |
| COVID19-Host(a)ge | 1610 | 2205 |
| deCODE | 89 | 274322 |
| Determining the Molecular Pathways and Genetic Predisposition of the Acute Inflammatory Process Caused by SARS-CoV-2 | 311 | 302 |
| FinnGen | 83 | 238628 |
| GEN-COVID | 571 | 2472 |
| Genes & Health | 62 | 27353 |
| Genetic determinants of COVID-19 complications in the Brazilian population | 756 | 1637 |
| Genetic modifiers for COVID-19 related illness | 109 | 1484 |
| Genetics of COVID-related Manifestation | 69 | 6500 |
| genomiCC | 1676 | 8380 |
| Penn Medicine Biobank | 66 | 8536 |
| Qatar Genome Program | 60 | 13360 |
| The genetic predisposition to severe COVID-19 | 78 | 3778 |
| UK Biobank | 413 | 420118 |

The data were obtained from the website <https://www.covid19hg.org/>.
