# Supplementary figures and images for: Modifiable lifestyle factors and severe COVID-19 risk: a Mendelian randomisation study
Source: BMC Med Genomics. 2021 Feb 3;14:38. doi: 10.1186/s12920-021-00887-1 (PMC7856619; doi:10.1186/s12920-021-00887-1)

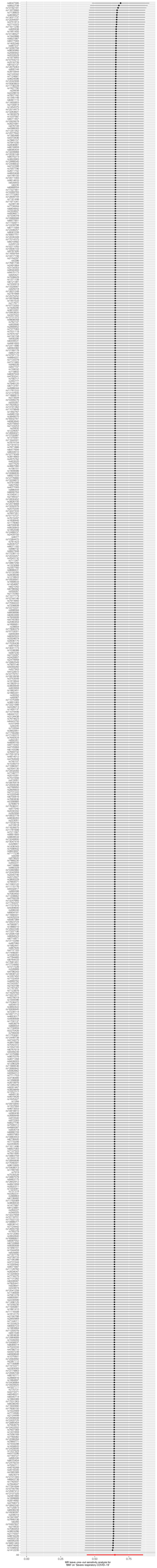

Supplement: Supplementary file 4 — Additional file 4: Supplementary Fig. 1. Leave-one-out analysis results for body mass index and severe respiratory COVID-19. [file 12920_2021_887_MOESM4_ESM.pdf]

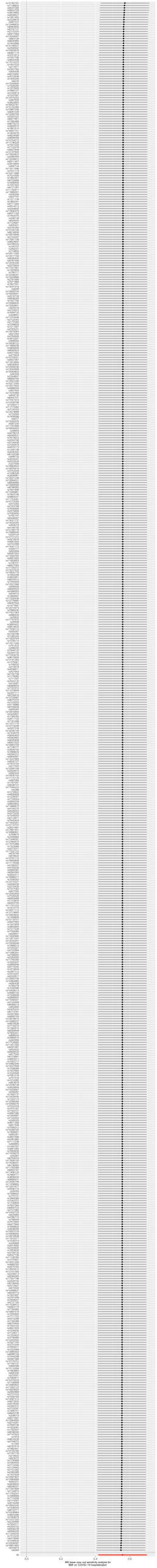

Supplement: Supplementary file 5 — Additional file 5: Supplementary Fig. 2. Leave-one-out analysis results for body mass index and COVID-19 hospitalization. [file 12920_2021_887_MOESM5_ESM.pdf]

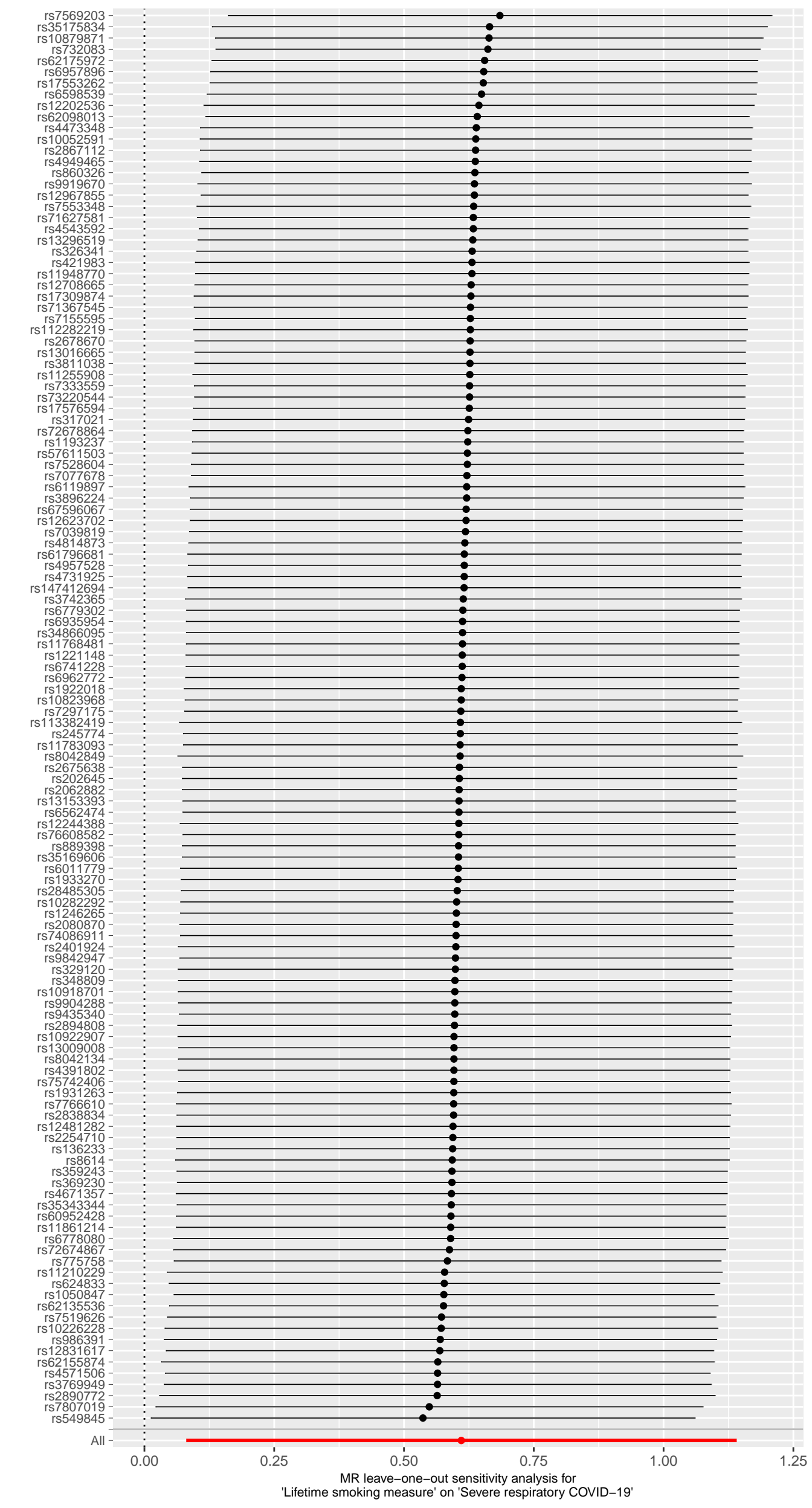

Supplement: Supplementary file 6 — Additional file 6: Supplementary Fig. 3. Leave-one-out analysis results for lifetime smoking and severe respiratory COVID-19. [file 12920_2021_887_MOESM6_ESM.pdf]

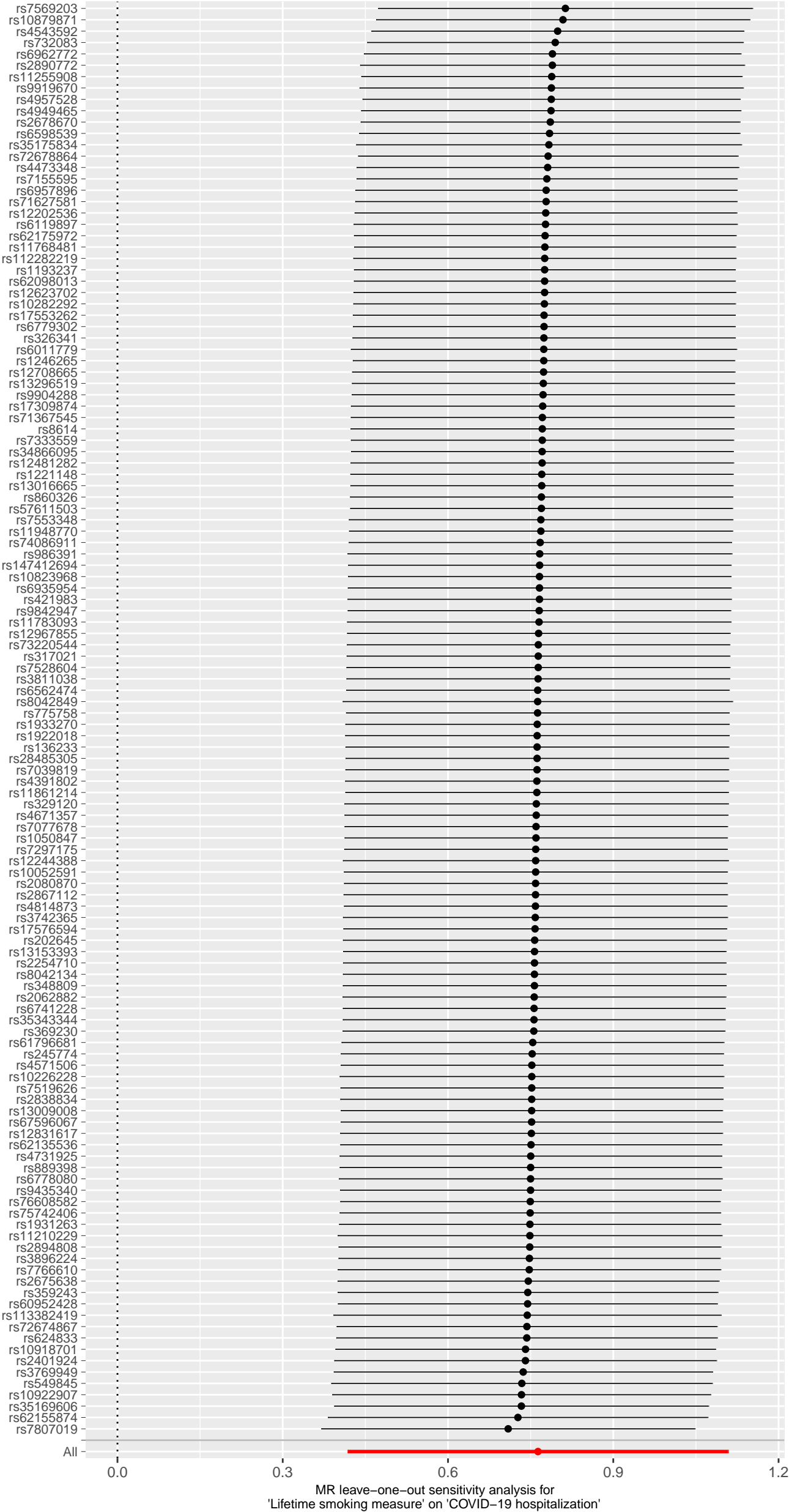

Supplement: Supplementary file 7 — Additional file 7: Supplementary Fig. 4. Leave-one-out analysis results for lifetime smoking and COVID-19 hospitalization. [file 12920_2021_887_MOESM7_ESM.pdf]

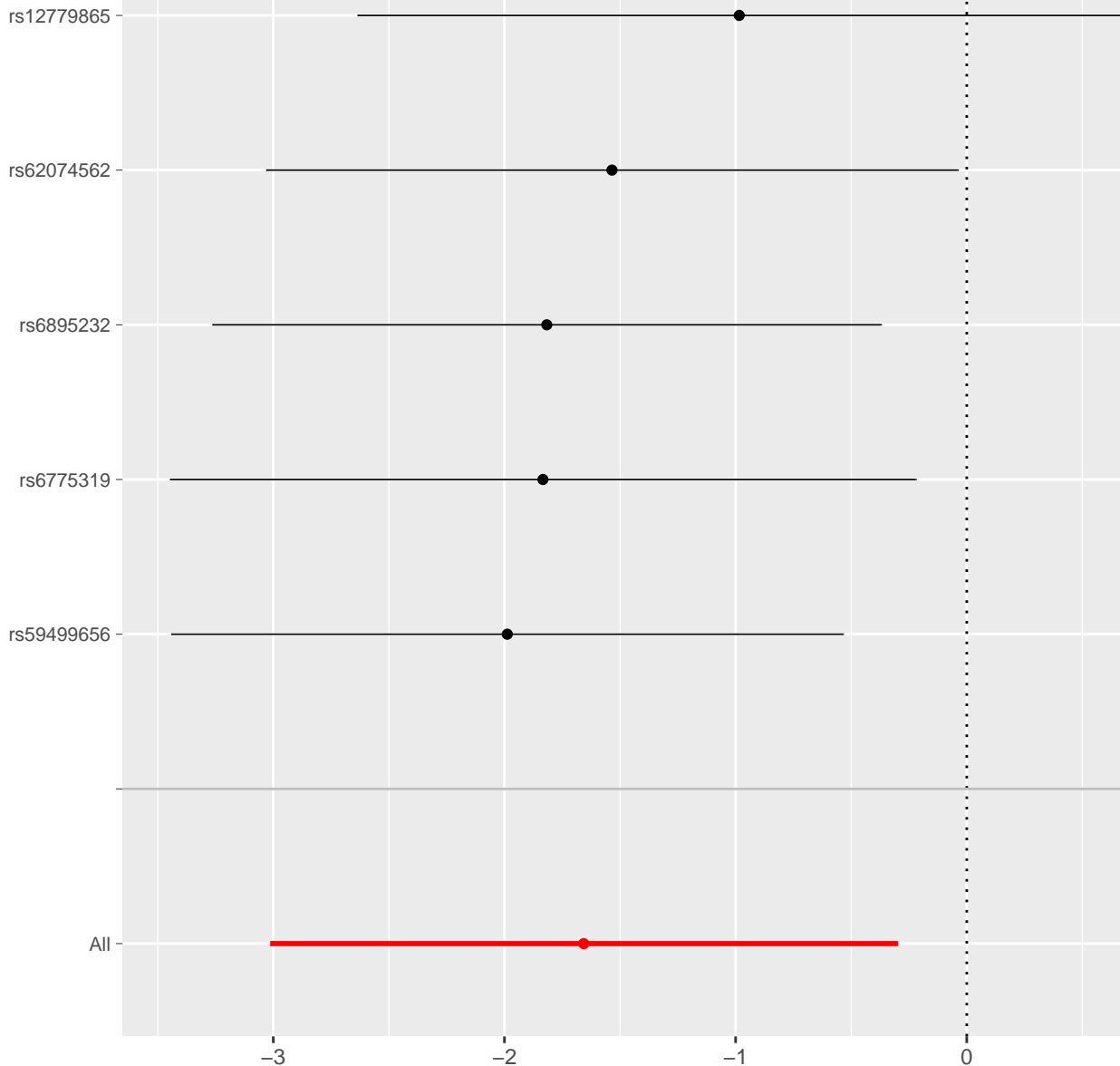

MR leave-one-out sensitivity analysis for  
'Physical activity' on 'Severe respiratory COVID-19'

Supplement: Supplementary file 8 — Additional file 8: Supplementary Fig. 5. Leave-one-out analysis results for physical activity and severe respiratory COVID-19. [file 12920_2021_887_MOESM8_ESM.pdf]

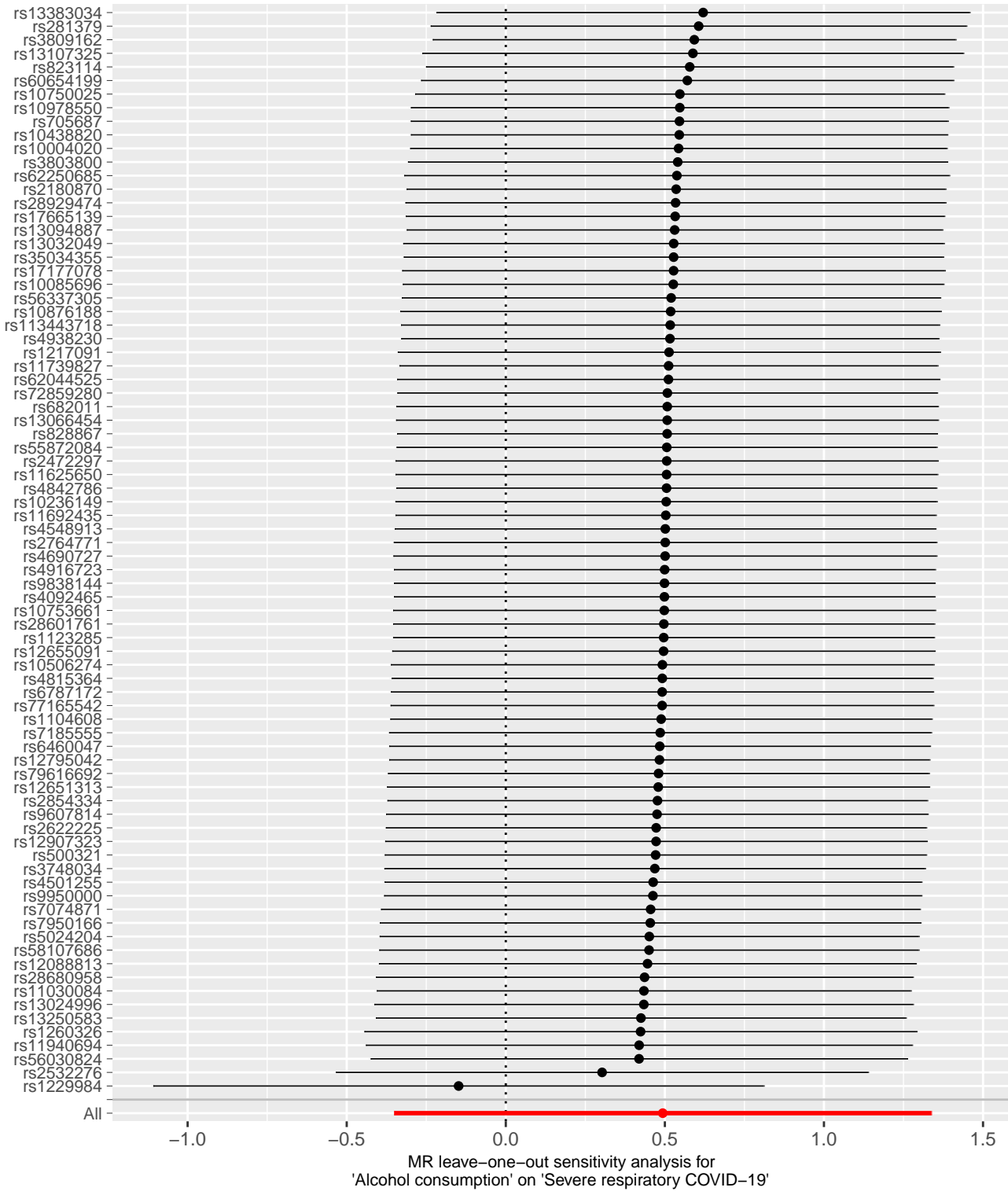

Supplement: Supplementary file 10 — Additional file 10: Supplementary Fig. 7. Leave-one-out analysis results for alcohol consumption and severe respiratory COVID-19 using all genetic variants. [file 12920_2021_887_MOESM10_ESM.pdf]

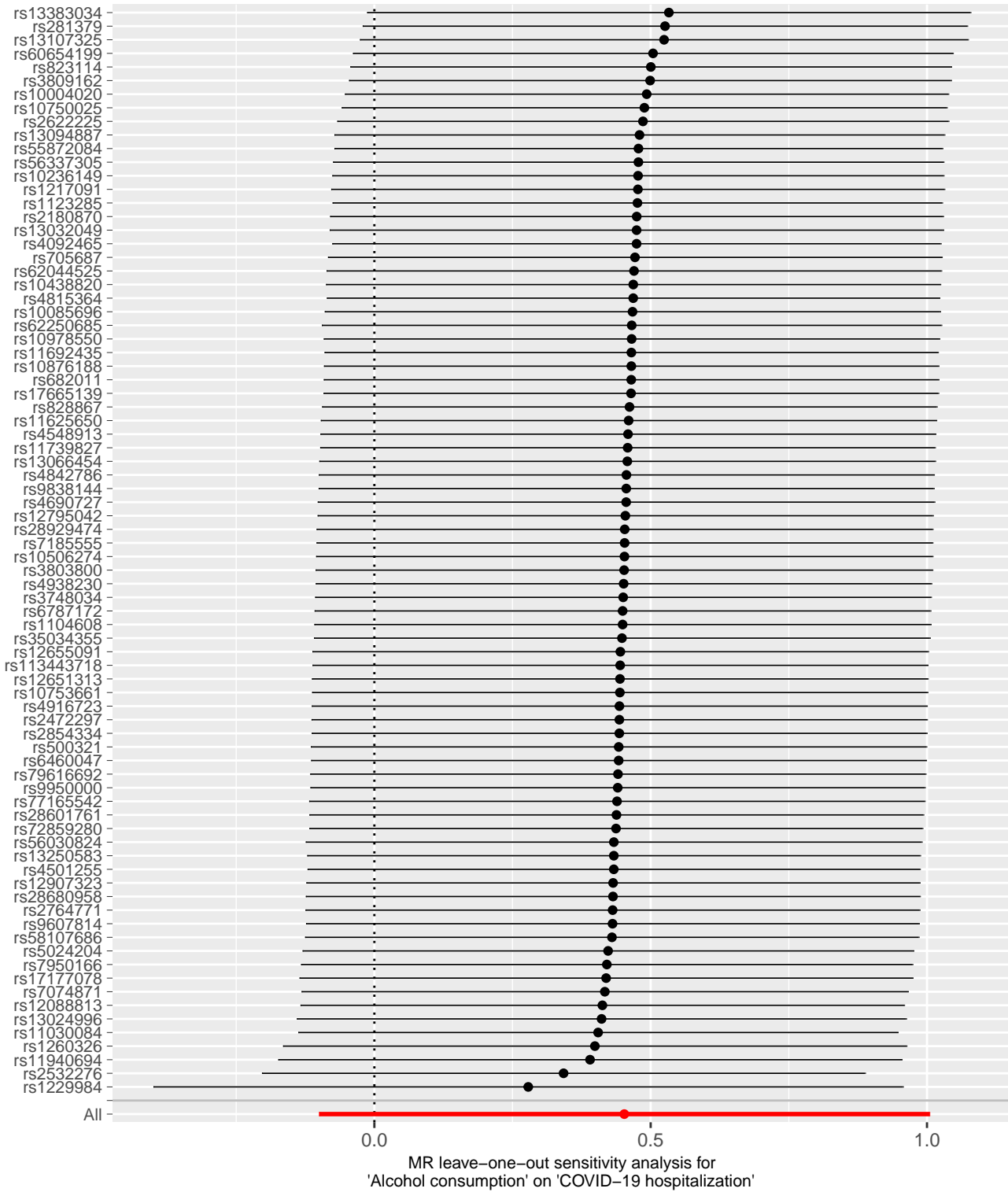

Supplement: Supplementary file 11 — Additional file 11: Supplementary Fig. 8. Leave-one-out analysis results for alcohol consumption and COVID-19 hospitalization using all genetic variants. [file 12920_2021_887_MOESM11_ESM.pdf]

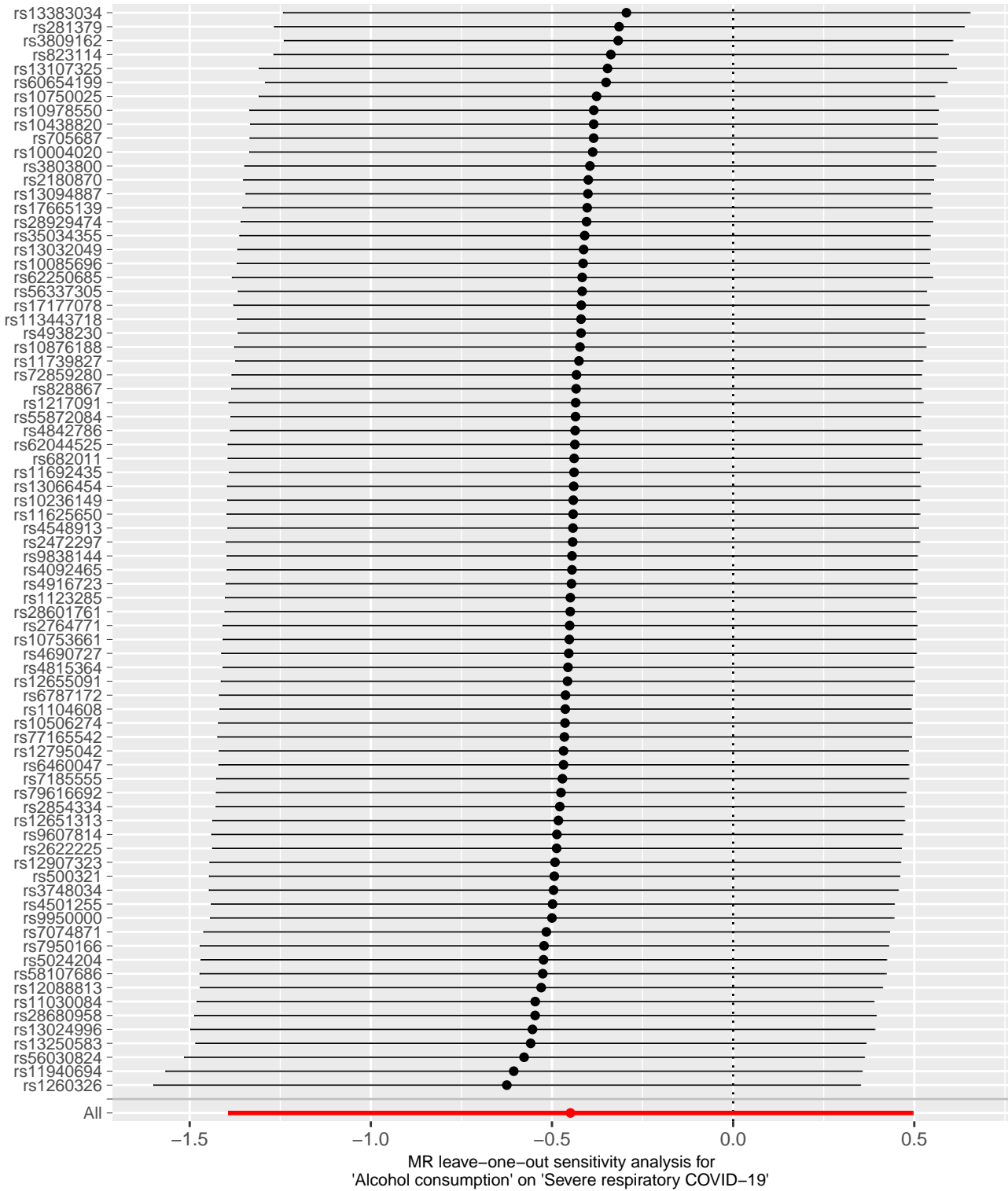

Supplement: Supplementary file 12 — Additional file 12: Supplementary Fig. 9. Leave-one-out analysis results for alcohol consumption and severe respiratory COVID-19 after removing rs1229984 and rs2532276. [file 12920_2021_887_MOESM12_ESM.pdf]

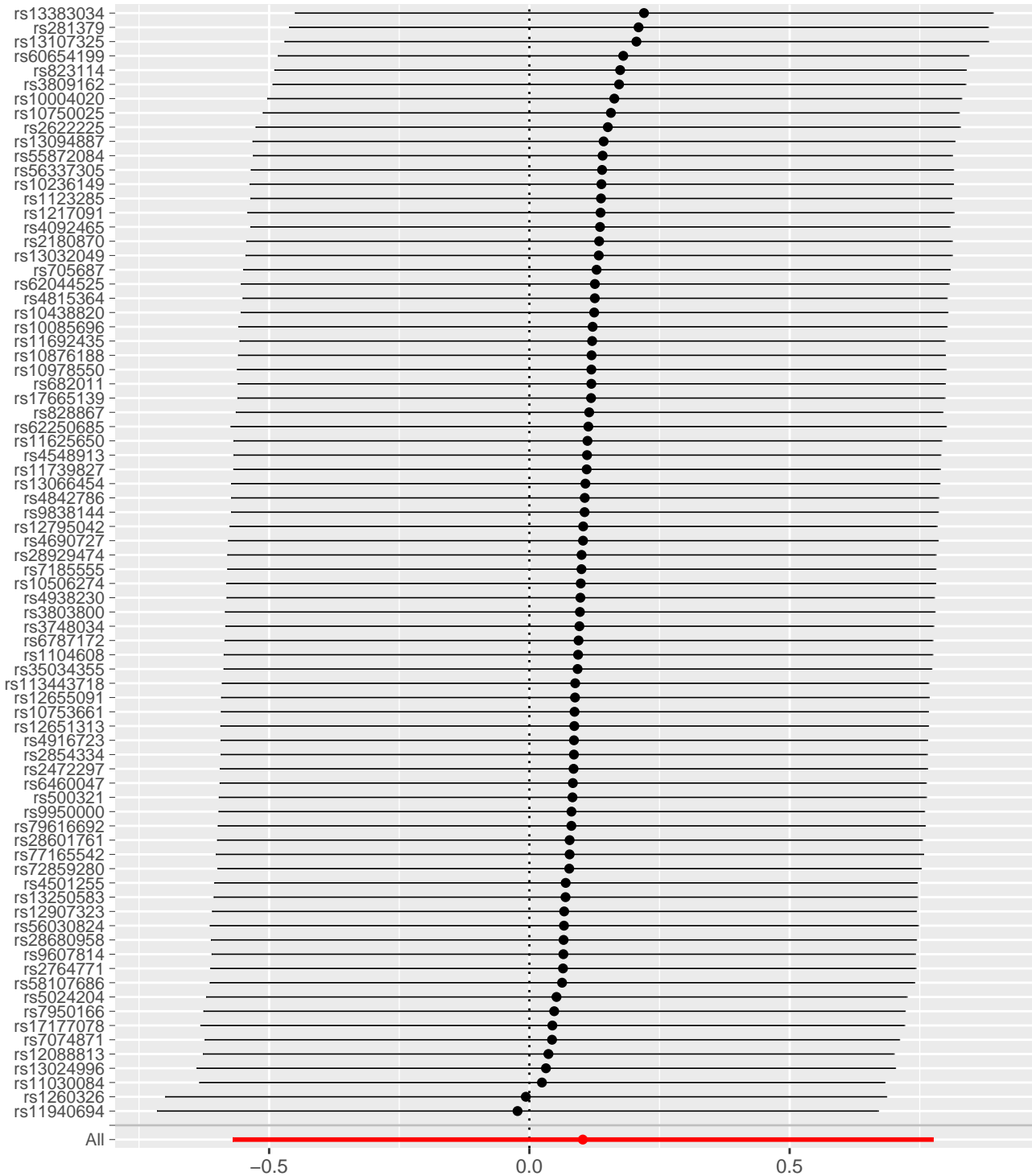

Supplement: Supplementary file 13 — Additional file 13: Supplementary Fig. 10. Leave-one-out analysis results for alcohol consumption and COVID-19 hospitalization after removing rs1229984 and rs2532276. [file 12920_2021_887_MOESM13_ESM.pdf]

# MR Method

- Inverse variance weighted
- MR Egger

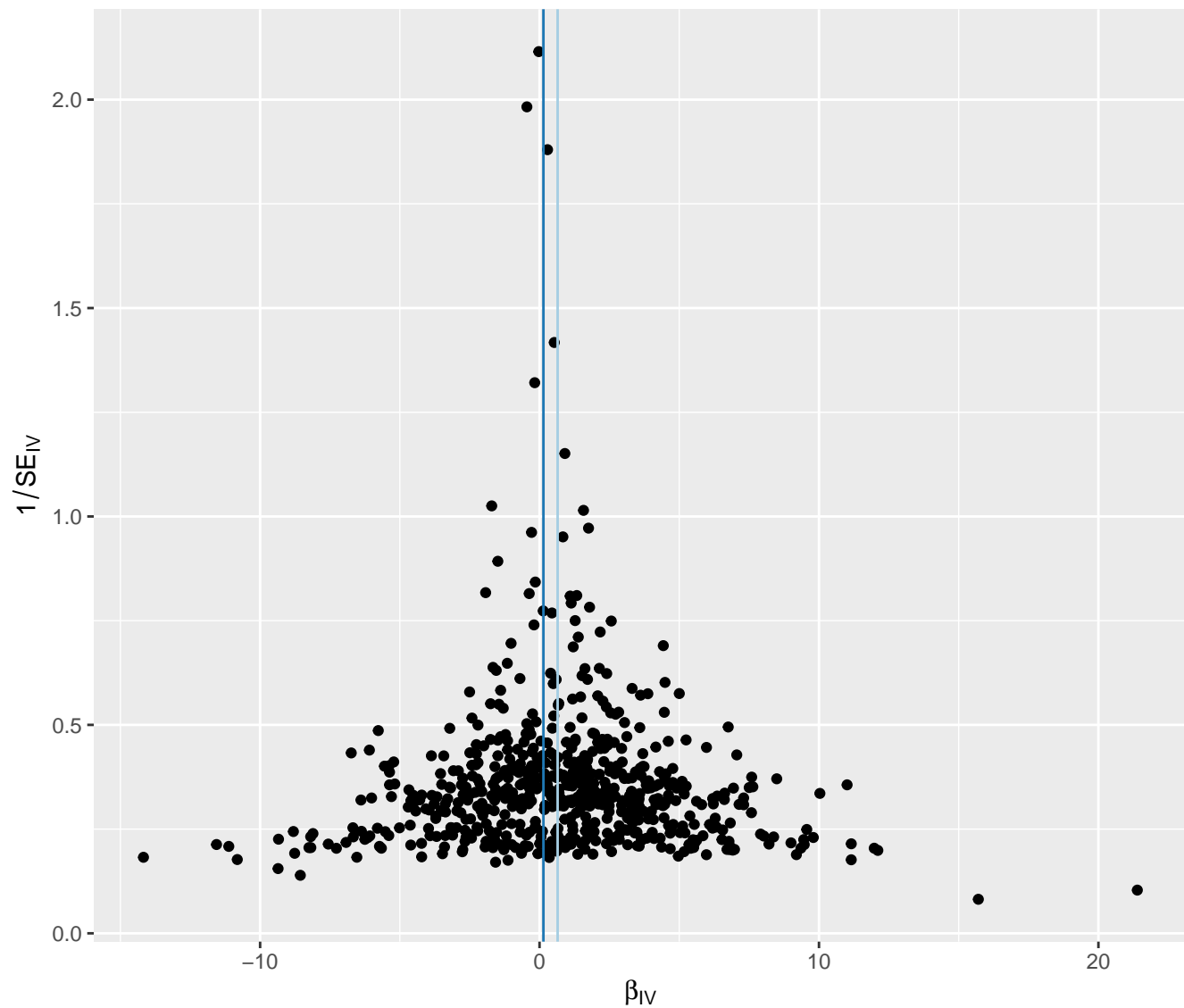

Supplement: Supplementary file 14 — Additional file 14: Supplementary Fig. 11. Funnel plot for body mass index and severe respiratory COVID-19. [file 12920_2021_887_MOESM14_ESM.pdf]

# MR Method

- Inverse variance weighted
- MR Egger

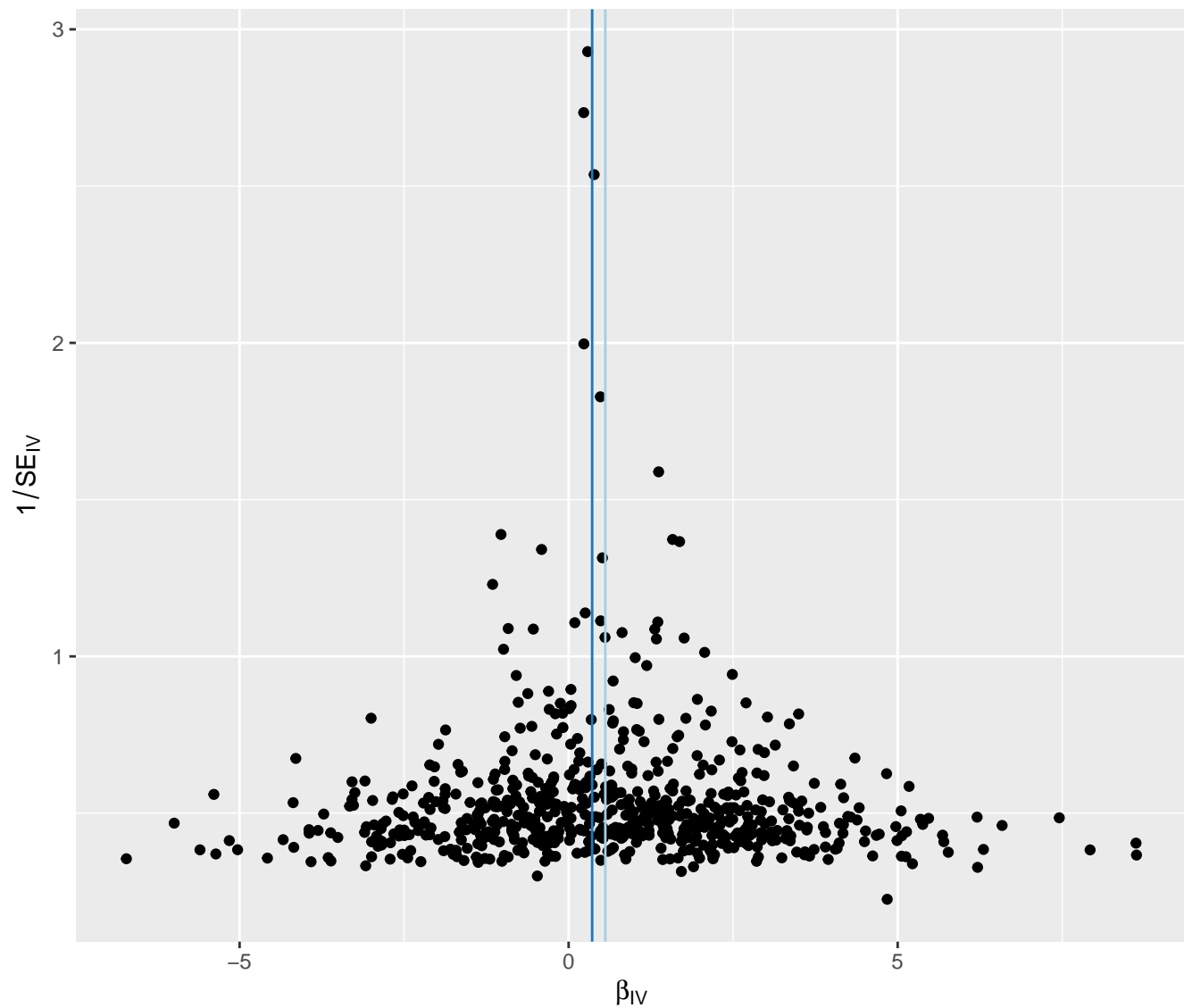

Supplement: Supplementary file 15 — Additional file 15: Supplementary Fig. 12. Funnel plot for body mass index and COVID-19 hospitalization. [file 12920_2021_887_MOESM15_ESM.pdf]

# MR Method

- Inverse variance weighted
- MR Egger

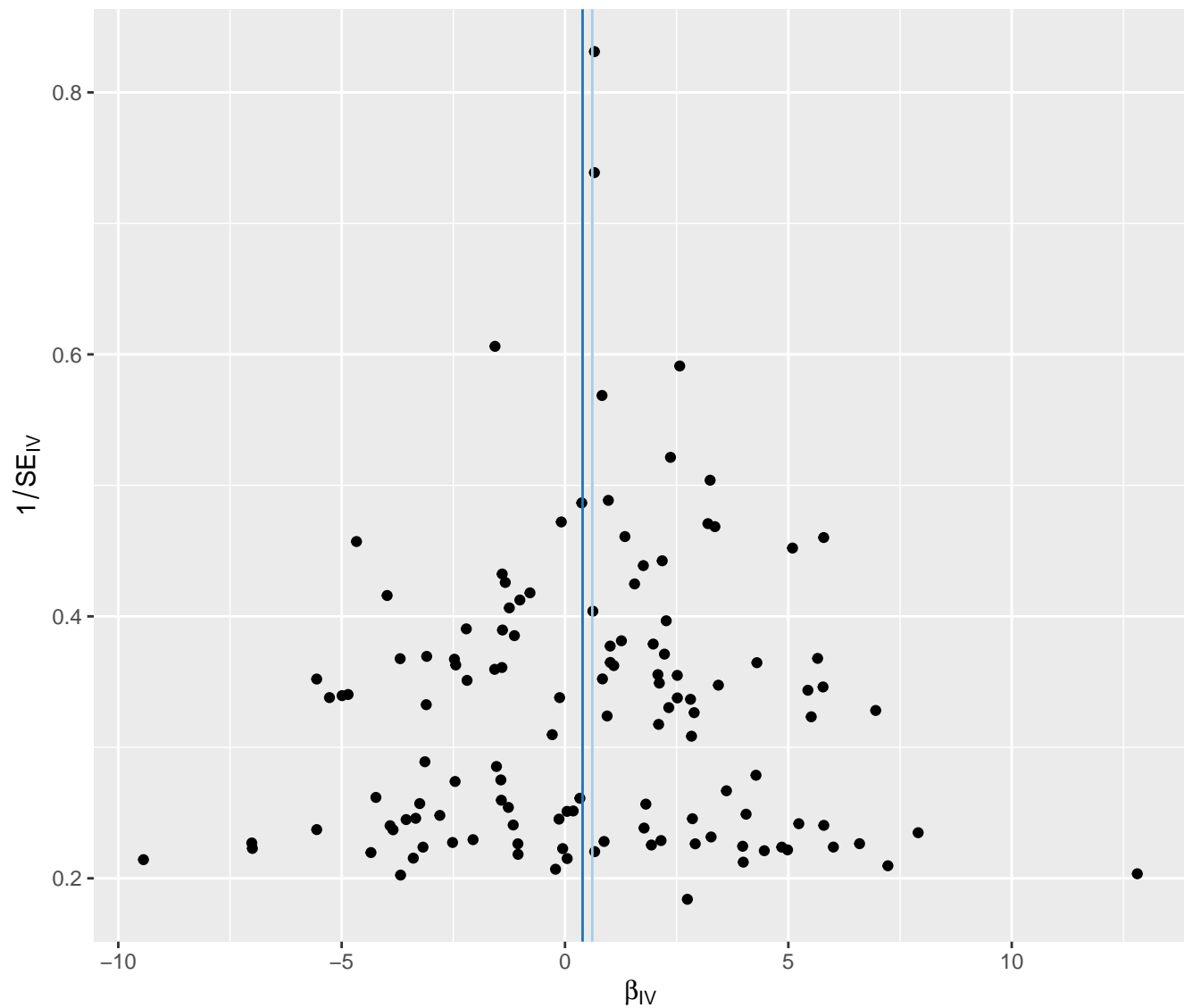

Supplement: Supplementary file 16 — Additional file 16: Supplementary Fig. 13. Funnel plot for lifetime smoking and severe respiratory COVID-19. [file 12920_2021_887_MOESM16_ESM.pdf]

# MR Method

- Inverse variance weighted
- MR Egger

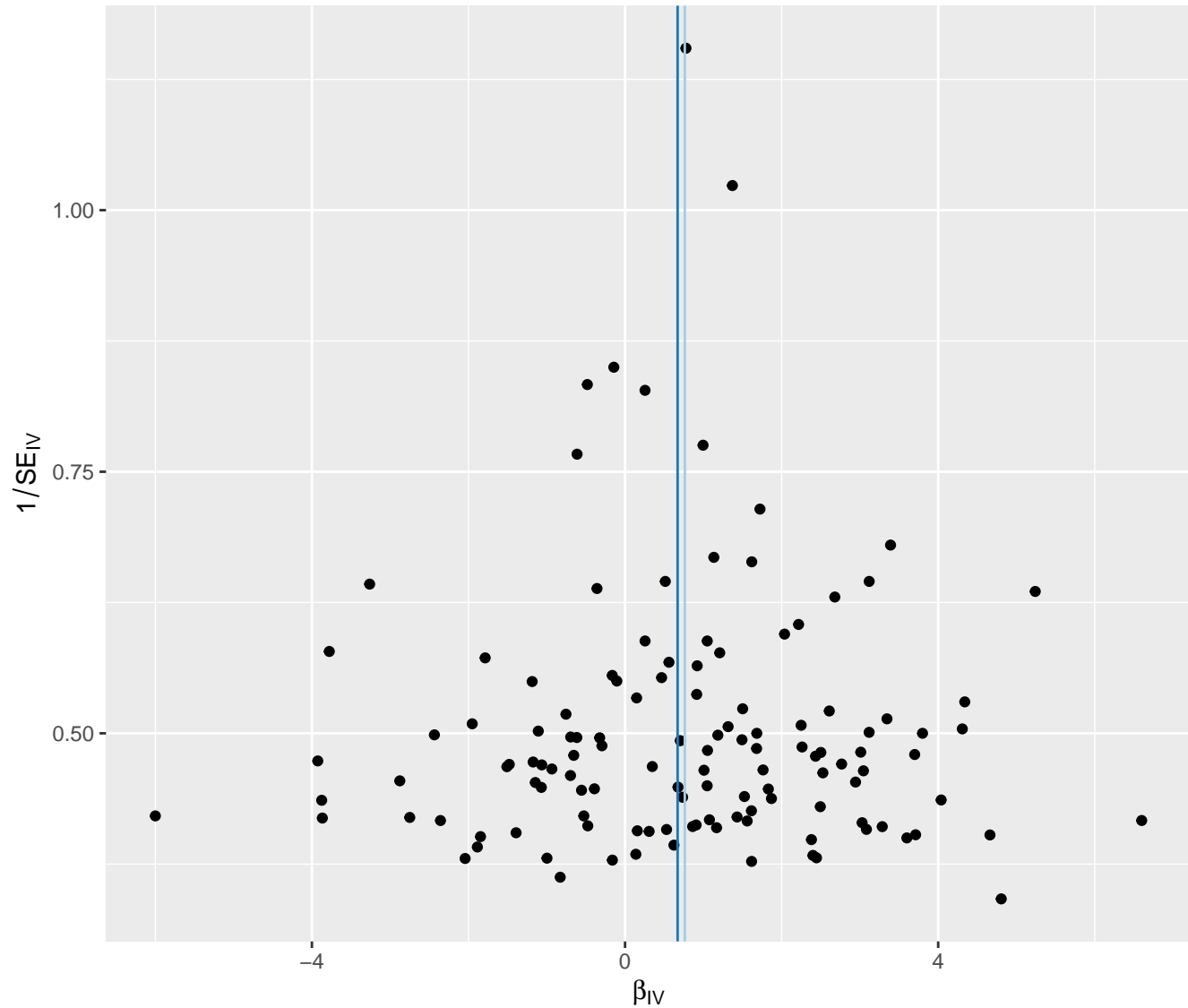

Supplement: Supplementary file 17 — Additional file 17: Supplementary Fig. 14. Funnel plot for lifetime smoking and COVID-19 hospitalization. [file 12920_2021_887_MOESM17_ESM.pdf]

# MR Method

- Inverse variance weighted
- MR Egger

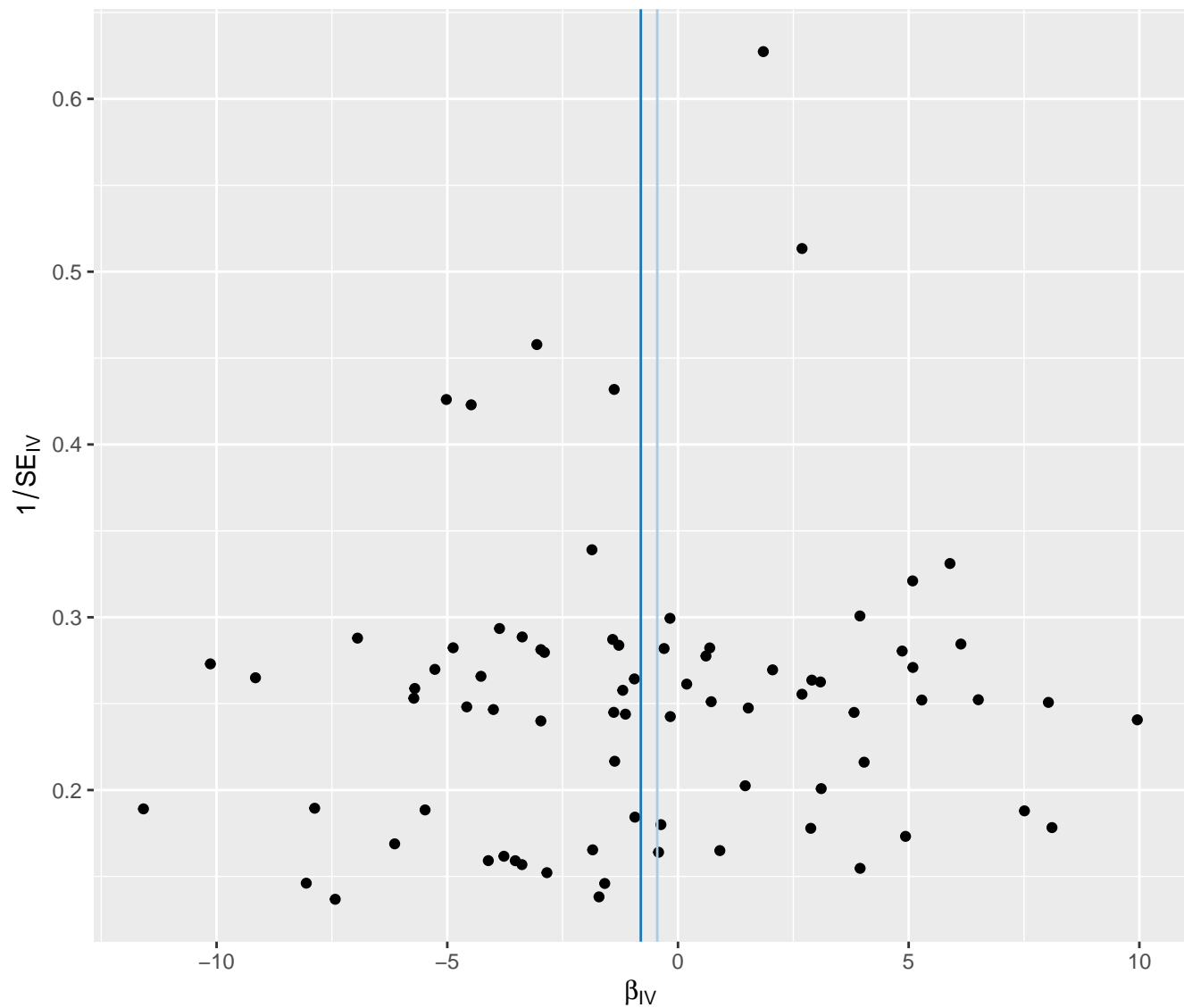

Supplement: Supplementary file 18 — Additional file 18: Supplementary Fig. 15. Funnel plot for alcohol consumption and severe respiratory COVID-19 after removing rs1229984 and rs2532276. [file 12920_2021_887_MOESM18_ESM.pdf]

# MR Method

- Inverse variance weighted
- MR Egger

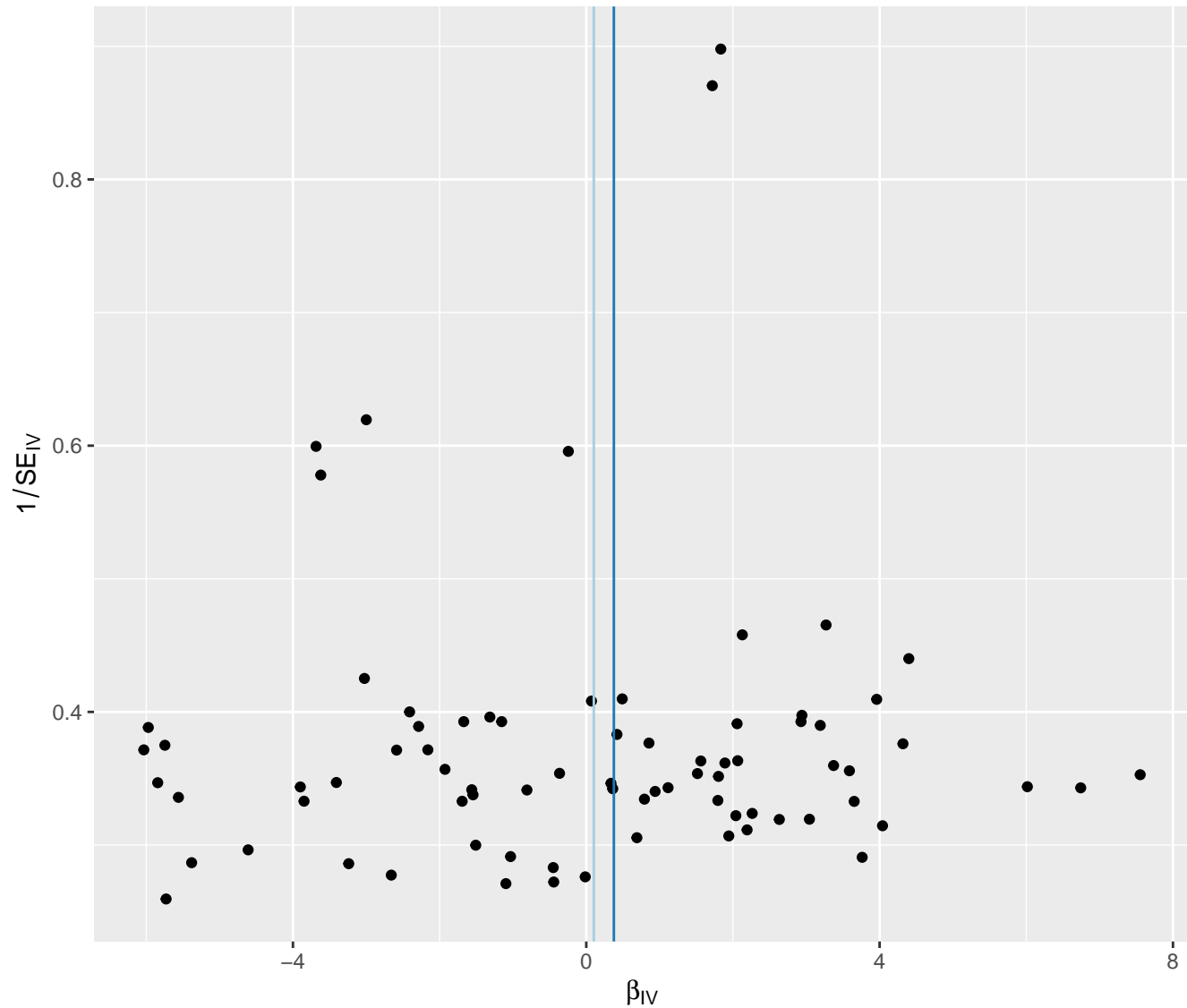

Supplement: Supplementary file 19 — Additional file 19: Supplementary Fig. 16. Funnel plot for alcohol consumption and COVID-19 hospitalization after removing rs1229984 and rs2532276. [file 12920_2021_887_MOESM19_ESM.pdf]

# MR Method

- Inverse variance weighted
- MR Egger

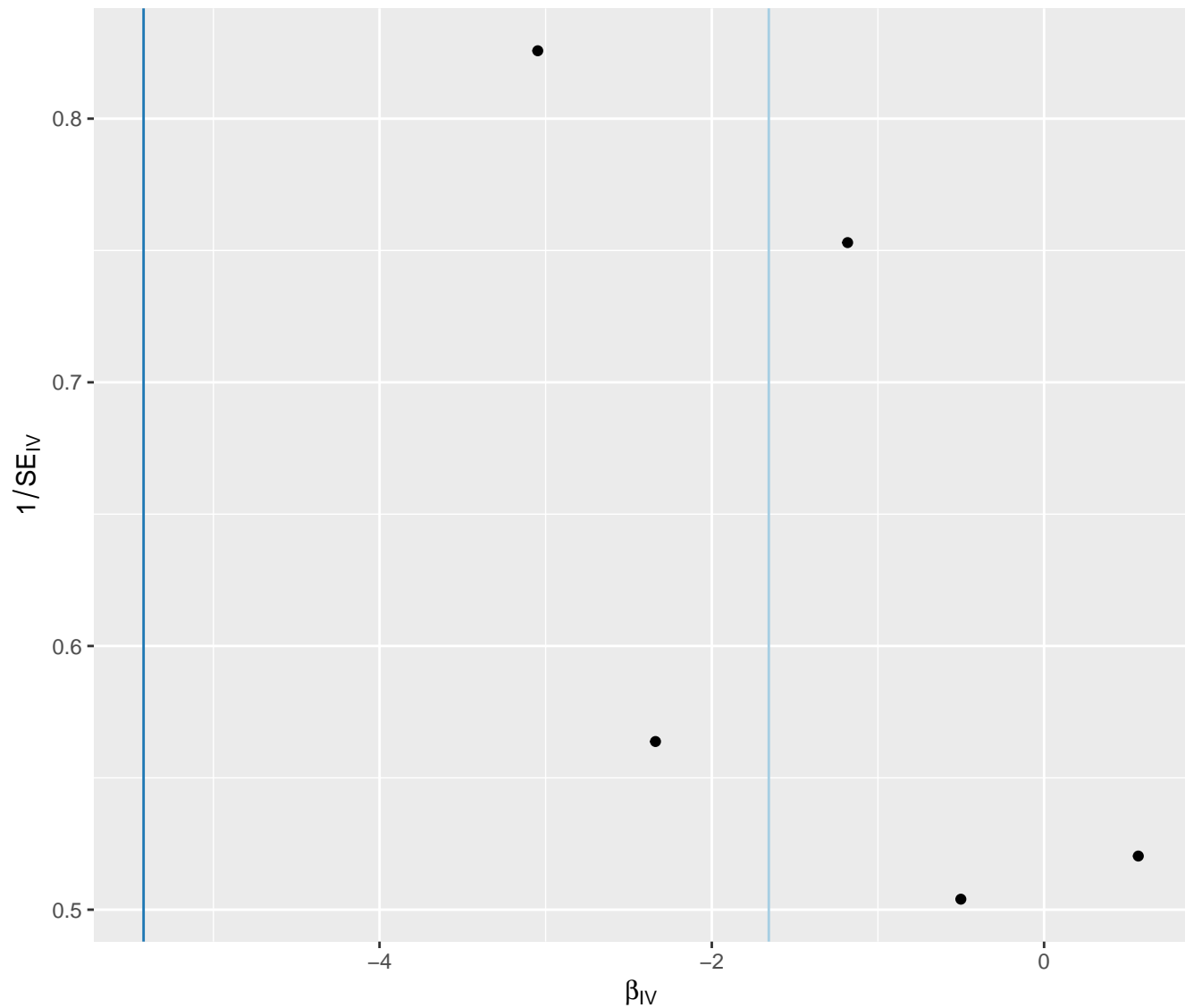

Supplement: Supplementary file 20 — Additional file 20: Supplementary Fig. 17. Funnel plot for physical activity and severe respiratory COVID-19. [file 12920_2021_887_MOESM20_ESM.pdf]

# MR Method

- Inverse variance weighted
- MR Egger

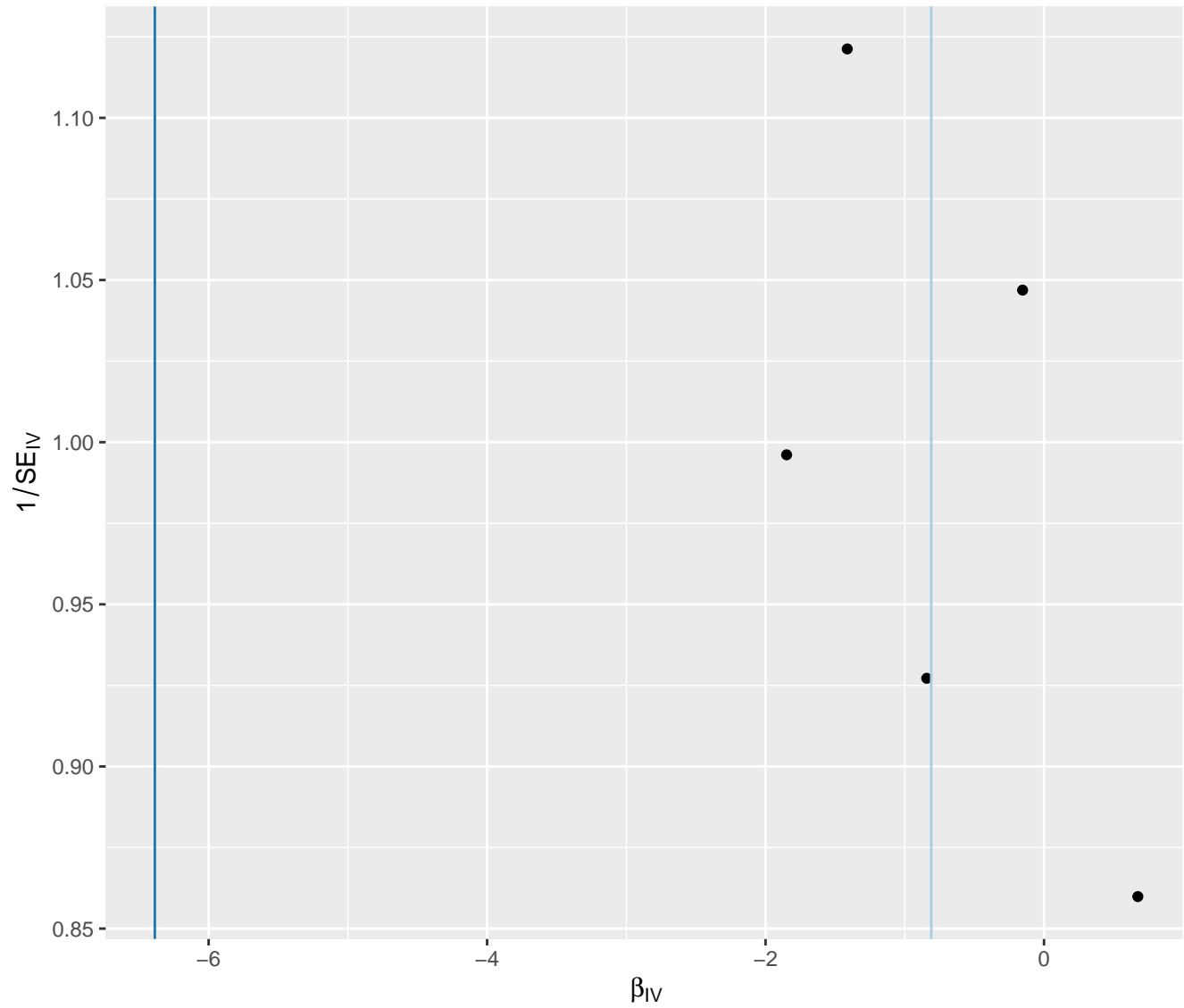

Supplement: Supplementary file 21 — Additional file 21: Supplementary Fig. 18. Funnel plot for physical activity and COVID-19 hospitalization. [file 12920_2021_887_MOESM21_ESM.pdf]
