# Supplementary material for: Modifiable lifestyle factors and severe COVID-19 risk: a Mendelian randomisation study
Source: BMC Med Genomics. 2021 Feb 3;14:38. doi: 10.1186/s12920-021-00887-1 (PMC7856619; doi:10.1186/s12920-021-00887-1)
Supplement: Supplementary file 9 — Additional file 9: Supplementary Fig. 6. Leave-one-out analysis results for physical activity and COVID-19 hospitalization. [file 12920_2021_887_MOESM9_ESM.pdf]

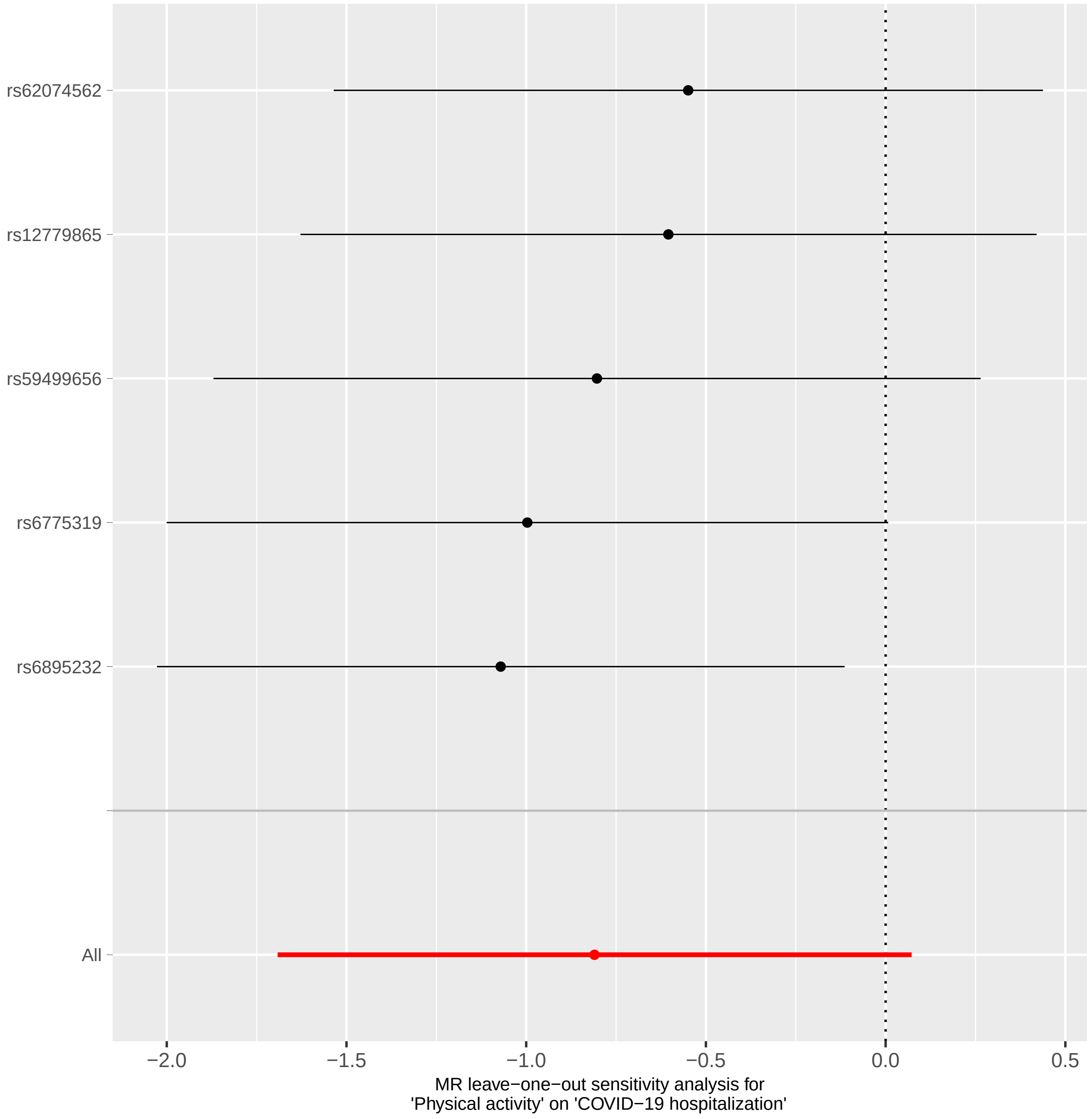
rs62074562
rs12779865
rs59499656
rs6775319
rs6895232
All
−2.0
−1.5
−1.0
−0.5
0.0
0.5
MR leave−one−out sensitivity analysis for
'Physical activity' on 'COVID−19 hospitalization'
